# Public opinions and attitudes toward a state monopoly: a study of the finnish gambling system

**DOI:** 10.1186/s12889-023-16917-9

**Published:** 2023-10-16

**Authors:** Joseph R. Macey, Brett L. Abarbanel, Sari Castrén, Juho J. Hamari, Anne H. Salonen

**Affiliations:** 1 Centre of Excellence in Game Culture Studies, University of Turku, Turku, Finland; 2 Gamification Group, Faculty of Information Technology and Communication Sciences, Tampere University, Room B2125, Pinni B, Kanslerinrinne 1, 33100 Tampere, Finland; 3 International Gaming Institute, University of Nevada, Las Vegas, NV USA; 4 William F. Harrah College of Hospitality, University of Nevada, Las Vegas, NV USA; 5 Finnish Institute for Health and Welfare, Helsinki, Finland; 6 University of Turku, Turku, Finland; 7 University of Helsinki, Helsinki, Finland; 8 University of Eastern Finland, Faculty of Health Sciences, Kuopio, Finland

**Keywords:** Gambling, Regulation, Public Opinion, Harm Reduction, ATGS, Monopoly

## Abstract

**Background:**

Gambling regulated through a state monopoly is often justified for reasons of public health, that is, that monopolies are a more effective means of reducing potential harm. This focus on harm prevention has increased in recent years, particularly as a result of pressures arising from the growth of online gambling and of legislation designed to promote competition. While prior works have examined the role of stakeholders in influencing policy decisions and in public discussions of the monopoly systems, attention has been focused on those with direct financial interests; the opinions of the public have largely been absent from these discussions. In 2017 Finland restructured its monopoly order to improve efficacy of addressing gambling related harms; this restructuring offers a valuable insight into public perceptions of and attitudes toward the suitability of the Finnish system to address gambling-related harm.

**Methods:**

This work uses Structural Equation Modelling and compares attitudes toward the Finnish system between 2015 (pre-restructuring) and 2019 (post-restructuring).

**Results:**

Overall public opinion of the Finnish system as being suitable for addressing gambling harms declined between 2015 and 2019, despite the restructuring. Several predictors of attitudes were identified, however, the majority had small effect sizes, while the model explained little variance.

**Conclusion:**

This work concludes that existing approaches to examining public opinions of gambling regulation should be amended to include additional predictors. Furthermore, it is likely that context-specific predictors should be included in models, in order to reflect the socio-cultural history of the population being investigated. Such predictors should be determined in respect to the population of interest but, for example, could include items measuring trust in authority, political orientation, cultural acceptance of gambling, or religious affiliation.

## Introduction

Where gambling is legal, it is regulated according either state monopoly, licensing regime, or a hybrid of the two. The dominant rationale for adopting a monopoly is that it facilitates greater ability to reduce harm associated with gambling while also serving as a means of generating tax revenues [1]. While evidence on the effectiveness of monopolies to reduce gambling-related harms is mixed, a recent review found that both the prevalence of problem gambling and total consumption of gambling is equal or lower under monopolies than under licencing regimes [2]. Furthermore, the focus on harm prevention has been strengthened in recent years, particularly as a result of pressures arising from the growth of online gambling and, specifically, EU competition regulations [3, 4].

As is common with gambling entities run by the state, controversy surrounds the apparently contradictory role of monopolies as both commercial provider of gambling products and services and as a funder of third sector, social organisations [5, 6]. Prior works have examined the role of stakeholders in influencing policy decisions in general [7], and in public discussions of the monopoly systems in particular [8]. However, one stakeholder is notably absent from these discussions: the public. Indeed, even those members of the public who have lived experience of gambling harm are only recently being incorporated as collaborators in policy design [9–12].

Alongside Norway, Finland is one of two countries which currently employ a full monopoly system in respect to the regulation of gambling. Until 2016, gambling in Finland was administered by three separate, state-controlled operators each of which were responsible for different markets. The system was reformed in 2017, with the three operators merged to form a single entity, Veikkaus Ltd., with the purpose of the merger being to eliminate competition between the three former gambling operators and to prevent and reduce gambling harms more effectively [1]. Further changes to the system have been suggested, with calls to limit the availability of slot machines in public spaces being increasingly common [13, 14]. In addition, there has been recent controversy surrounding the role of Veikkaus as a state-owned commercial provider of gambling products and services and the apparent lack of consideration given to the potential social harms that result [15].

Finland is currently considering moving away from a full monopoly and opening the market to foreign online operators, as has been the case in many Western European countries over the past two decades [16–18]. Given the relatively high rates of gambling participation in Finland, both historic and contemporary [19], the recent restructuring of the state monopoly system offers a valuable insight into perceptions of and attitudes toward this form of regulation among an engaged public.

The aim of this work, therefore, is to conduct exploratory research which identifies predictors of public attitudes toward both the Finnish monopoly system in general, and a specific policy aimed at reducing access to gambling. With Finland considering a dismantling of its monopoly, this work offers a timely insight into public attitudes toward gambling regulation and a view toward potential public response to major operational and regulatory change in the Finnish gambling market. It is expected that this work will provide lessons which can inform the approach of other bodies contemplating regulatory change, for example in response to the legalisation of previously prohibited substances [20] or the expansion of gambling into new domains [21].

### Research model

Prior research identifies individually-held attitudes toward gambling as the main predictor of predicting attitudes to how gambling is regulated [22, 23]. Thus, this research model incorporates such attitudes, as well as appraisals of different regulatory frameworks.

**Fig. 1:**
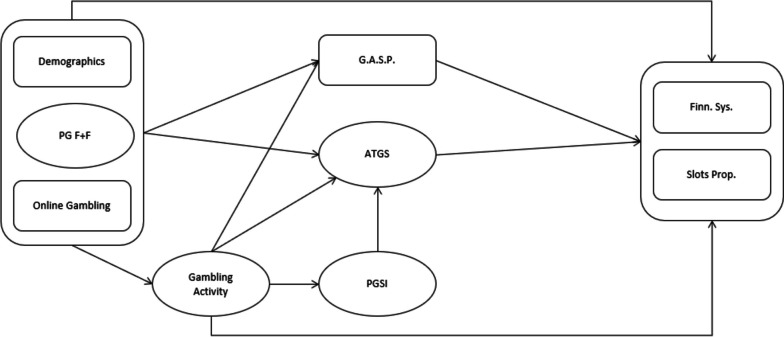
Research model

A simplified representation of the research model is provided in Fig. 1 (below). Opinion of the suitability of the Finnish system to address gambling harms and opinion of the slot machine proposal will form the two dependent variables (DVs). Two variables measuring attitudes to gambling will mediate the relationships between these two DVs and all independent variables (IVs). Mediating variables are the Attitude Towards Gambling Scale (ATGS-8) and a single scale item recording opinion on the seriousness of gambling problems in Finland.

Three IVs comprise Age, Gender, and Past-12-month Online Gambling Participation: both age and gender have been found to influence attitudes toward gambling [24], while those who gamble online have been found to exhibit more positive attitudes toward gambling than those who do not [25]. Furthermore, intergroup theory suggests that positive contact with gambling engenders positive attitudes and vice versa [24], contact with gambling will be assessed via three separate variables, the first of which is experiences of gambling problems among friends and family. The remaining two IVs reflect personal experiences of gambling and are recorded using gambling activity and problem gambling assessment, measured via the Problem Gambling Severity Index (PGSI).

Given the aim of the research is to investigate potential changes in opinions as a result of the merger, we formulate hypotheses related to dependent variables only. Accordingly, we hypothesise that:

- H1. Negative opinion of the Finnish state monopoly system is expected to be associated with: a) positive attitudes to gambling; b) younger age; c) higher rates of problem gambling among friends and family; d) male gender; e) prior participation in online gambling; f) increased gambling activity; g) increased PGSI score.
- H2. Opposition to the proposal to limit access to slot machines is expected to be associated with: a) positive attitudes to gambling; b) younger age; c) lower rates of problem gambling among friends and family; d) male gender; e) prior participation in online gambling; f) increased gambling activity; g) increased PGSI score.
- H3. As a result of the 2017 merger, approval of both a) the proposal to reduce access to slot machines and b) overall opinion of the Finnish system will be higher in 2019 than in 2015.

## Methods

### Participants and procedure

This study utilises two sets of data collected in the Finnish Gambling Surveys (FGS) of 2015 and 2019 [26]. The FGS is conducted every four years and is a nationally-representative, cross-sectional sample. These two surveys were chosen as they lie either side of the 2017 reform of the state-run monopoly system. The surveys were commissioned by the Finnish Ministry of Social Affairs and Health and were conducted by Statistics Finland via computer-assisted telephone interviews. Eligible respondents were Finnish nationals aged 15–74, who resided in mainland Finland, and were native speakers of either Finnish, Swedish, or Sami with respondents being able to select from either Finnish or Swedish when completing the survey. Systematic random sampling was used to select potential participants from the National Population Register’s sampling frame. The response rates for 2015 and 2019 were 62% and 52%, respectively; prior to analysis outliers were identified by examining z scores, those with values of ± 3.29 were removed from both datasets [27].

### Measures

The short form Attitudes Towards Gambling Scale (ATGS-8 [28, 29]) measures attitudes to gambling in general, rather than any specific activity, and has been designed to be used across distinct populations using a five-point Likert scale (1 = strongly agree to 5 = strongly disagree). The eight items included in the short-form version address societal benefits and harms, individual experiences, and more generalised statements. Items are summed, with a higher score indicating more negative attitudes to gambling. For the purposes of this research scores were reverse coded in order that higher scores describe more positive attitudes. Attitudes to gambling were also assessed using the following separate question: “*Do you think gambling problems are a serious problem in Finland*?” (variable *G.A.S.P.*).

Gambling activity was measured by recording frequency of participation across individual activities and average weekly spend (€); frequency was measured using an ordinal categorical scale which, for the purpose of this study, was converted into an interval scale using coded midpoints [30]. The Problem Gambling Severity Index (PGSI; [31], is a nine-item measure for assessing problematic gambling behaviour and potential negative consequences, responses were recorded using a four-point Likert scale (0 = never to 3 = almost always). Items are summed, with higher scores indicating increased likelihood of problematic gambling behaviours.

Seven items measured the presence of gambling problems among participants’ friends and family. Finally, online gambling in the prior 12 months was recorded using a binary (yes/no) item; age and gender were also captured.

**Fig. 2:**
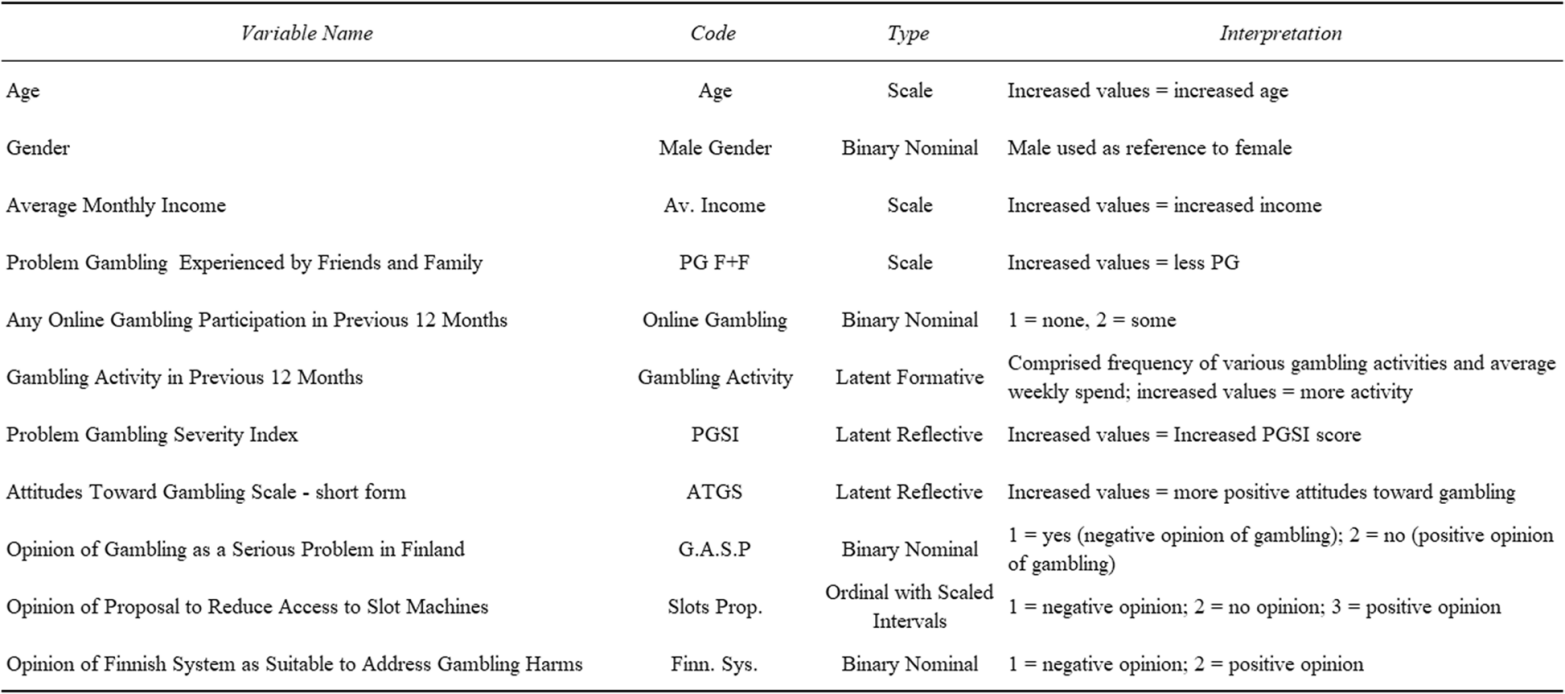
Guide to variables

Dependent variables assessing opinion of the Finnish monopoly system, “*Finn Sys.*” was measured with binary (yes/no) question, while opinion of a proposal to limit slot machine availability to dedicated gambling venues “*Slots Prop.*” was measured via an ordinal variable with scaled intervals. All variables included in the model are described in Fig. 2 (above) in order to aid interpretation.

This research is an informed exploratory look at identifying predictors of attitudes toward the Finnish system and a specific potential policy. This research model incorporates both formative latent variables, continuous variables, and binary categorical independent and dependant variables in a relatively complex model. As such, Partial Least Squares Structural Equation Modelling (PLS-SEM) was deemed to be the most suitable analytical method [32]. Given that the aim of the research was to investigate potential differences between two distinct time points (2015 and 2019), the multigroup analysis (MGA) function of SmartPLS 4 was used. MGA allows moderation effects to be understood across multiple relationships of a research model while accounting for a heterogenous population [33, 34]. MGA was deemed appropriate as this research used only two datasets which met the assumptions of normal population distribution and approximately equal size (ratio of 1.13) [35, 36].

### Role of the funding sources

The sources by which this work was funded had no role in the study design, analysis, or interpretation of the results of the manuscript or any phase of the publication process.

## Results

*ATGS* and *PGSI* were operationalised as single items; *Gambling Experiences: Friends and Family* and *Gambling Activity* are formative constructs, construct validity was therefore assessed using Outer VIF values, the highest value of either was 2.093. All assumptions were met and partial measurement invariance was confirmed, meaning that multigroup analysis could be performed [33], all tests were one-tailed.

Of the 2015 sample (*n* = 4,505), 51.1% reported male gender. Ages ranged from 15 to 74, with the mean being 47.5. The majority of respondents were married (49.9%) and in full-time employment (42%). Of the 2019 sample (*n* = 3,978), 50.8% reported male gender. Ages ranged from 15 to 74, with the mean age being 48.89. The majority of respondents were married (48.2%) and in full-time employment (43.9%).

When presenting results related to H1, opinion of the Finnish system, what is most evident is the lack of consistency. Only two independent variables, *Gender (Male)* and *Gambling Activity*, were found to have statistically significant, negative relationships for both 2015 and 2019. See Table 1, rows H1d and H1f, respectively.

**Table 1:** Summary of hypotheses and results

Code		Hypothesis	Supported?	Results		MGA analysis		
				2015	2019	Difference (2015—2019)	Direction of change	sig. (*p*)
H1	a	More positive attitudes to gambling (*ATGS*) are expected to be associated with negative opinions of opinion of Finnish monopoly system as a means of reducing harm (*Finn. Sys.*)	2019 only	*ATGS*—> *Finn. Sys.*: β = -.002, *p* = .325	*ATGS*—> *Finn. Sys.*: β = -.018, *p* = .006**	.017	negative	.043*
		More positive attitudes to gambling (*GASP*) are expected to be associated with negative opinions of opinion of Finnish monopoly system as a means of reducing harm (*Finn. Sys.*)	2015 only	*G:A:S:P*—> *Finn. Sys.*: β = -.025, *p* < .017*	*G:A:S:P*—> *Finn. Sys.*: β = -.020, *p* = .119	.006	positive	0.391
	b	A positive assessment of the Finnish system (*Finn. Sys.*) is expected to be associated with increased age (*Age*)	2015 only	*Age*—> *Finn. Sys.*: β = .033, *p* = . < .001***	*Age*—> *Finn. Sys.*: β = .009, *p* = .080	.025	negative	.001**
	c	A negative assessment of the Finnish system (*Finn. Sys.*) is expected to be associated with higher rates of problem gambling among friends and family (*PG F* + *F*)	2015 only	*PG F* + *F*—> *Finn. Sys.*: β = -.031, *p* < .001***	*PG F* + *F*—> *Finn. Sys.*: β = -.008, *p* = .146	.023	positive	.010*
	d	Negative assessment of the Finnish system (*Finn. Sys.*) is expected to be associated with males (*Male Gender*)	yes	*Male Gender*—> *Finn. Sys.*: β = -.093, *p* < .001***	*Male Gender*—> *Finn. Sys.*: β = -.049, *p* < .001***	.044	positive	.034*
	e	Negative assessment of the Finnish system (*Finn. Sys.*) is expected to be associated with prior participation in online gambling (*Online Gambling*)	2019 only	*Online Gambling*—> *Finn. Sys.*: β = -.020, *p* = .123	*Online Gambling*—> *Finn. Sys.*: β = -.063, *p* < .001***	.043	negative	.027*
	f	Negative assessment of the Finnish system (*Finn. Sys.*) is expected to be associated with increased gambling activity (*Gambling Activity*)	yes	*Gambling Activity*—> *Finn. Sys.*: β = -.047, *p* < .001***	*Gambling Activity*—> *Finn. Sys.*: β = -.029, *p* = .001**	.018	positive	0.072
	g	Negative assessment of the Finnish system (*Finn. Sys.*) is expected to be associated with increased PGSI score (*PGSI*)	2019 only	*PGSI*—> *Finn. Sys.*: β = .000, *p* = .401	*PGSI*—> *Finn. Sys.*: β = -.002, *p* = .012*	.002	negative	.042*
H2	a	More positive attitudes to gambling (*ATGS*) are expected to be associated with negative opinions of proposals to limit the availability of slot machines (*Slots Prop.*)	yes	*ATGS*—> *Slots Prop.*: β = -.410, *p* < .001***	*ATGS*—> *Slots Prop.*: β = -.404, *p* < .001***	.006	positive	0.386
		More positive attitudes to gambling (*GASP*) are expected to be associated with negative opinions of proposals to limit the availability of slot machines (*Slots Prop.*)	yes	*G:A:S:P*—> *Slots Prop.*: β = -.297, *p* < .001***	*G:A:S:P*—> *Slots Prop.*: β = -.318, *p* < .001***	.021	negative	0.333
	b	Support for the proposal to limit access to slot machines (*Slots Prop.*) are expected to be associated with increased age (*Age*)	yes	*Age*—> *Slots Prop*.: β = .112, *p* < .001***	*Age*—> *Slots Prop.*: β = .026, *p* = .005**	.086	negative	< .001***
	c	Support for the proposal to limit access to slot machines (*Slots Prop.*) are expected to be associated with higher rates of problem gambling among friends and family (*PG F* + *F*)	yes	*PG F* + *F*—> *Slots Prop.*: β = .084, *p* < .001***	*PG F* + *F*—> *Slots Prop.*: β = .044, *p* = .008**	.04	negative	< .001***
	d	Opposition to the proposal to limit access to slot machines (*Slots Prop.*) is expected to be associated with males (*Male Gender*)	yes	*Male Gender*—> *Slots Prop.*: β = -.324, *p* < .001***	*Male Gender*—> *Slots Prop.*: β = -.172, *p* < .001***	.152	positive	< .001***
	e	Opposition to the proposal to limit access to slot machines (*Slots Prop.*) is expected to be associated with prior participation in online gambling (*Online Gambling*)	yes	*Online Gambling*—> *Slots Prop.*: β = -.135, *p* = .001 **	*Online Gambling*—> *Slots Prop.*: β = -.120, *p* < .001***	.016	positive	0.391
	f	Opposition to the proposal to limit access to slot machines (*Slots Prop.*) is expected to be associated with increased gambling activity (*Gambling Activity*)	yes	*Gambling Activity*—> *Slots Prop*.: β = -.072, *p* < .001***	*Gambling Activity*—> *Slots Prop.*: β = -.079, *p* < .001***	.007	negative	0.25
	g	Opposition to the proposal to limit access to slot machines (*Slots Prop.*) are expected to be associated with increased PGSI score (*PGSI*)	no	*PGSI*—> *Slots Prop.*: β = .050, *p* < .001***	*PGSI*—> *Slots Prop.*: β = .055, *p* < .001***	.005	positive	0.347
H3	a	Approval of the proposal to reduce access to slot machines (*Slots Prop.*) will be higher in 2019 than in 2015	yes	Approval *Slots Prop.*: 2015 = 37.2% < 2019 = 52.3%				
	b	Overall opinion of the Finnish system (*Finn. Sys.*) will be higher in 2019 than in 2015	no	Approval *Finn. Sys.*: 2015 = 77.8% > 2019 = 71.9%				

Legend:

^*^significant at *p* = 0.05

^**^significant at *p* < 0.01

^***^significant at *p* < .001

Independent variables *G.A.S.P*, *PG F* + *F*, and *Age*, were found to have statistically significant relationships to opinion of the Finnish System in 2015 only; the first two of which were negative and the latter positive. See Table 1, rows H1a, H1c, and H1b, respectively.

Independent variables *ATGS*, *Online Gambling*, and *PGSI* all displayed statistically significant relationships to opinion of the Finnish System in 2019 only. See Table 1, rows H1a, H1e, and H1g, respectively.

All predictors had negligible effects on the dependent variable *Finn Sys.*, with the largest path coefficient (β) being -0.093 and the smallest -0.002. In addition, statistically significant differences between opinion of the Finnish system in 2015 and 2019 datasets were observed for: *ATGS*, *PG F* + *F*, *Gender (male)*, *Age*, *Online Gambling*, and *PGSI*. In the first three cases the opinion became more positive, while in the final three cases opinion became more negative. See Table 1 for full details.

In respect to H2, opinion of proposal to limit access to slot machines, results were much more consistent, with all independent variables displaying statistically significant relationships of the same direction in both 2015 and 2019. Of these, *ATGS*, *G.A.S.P.*, *Gender (male)*, *Online Gambling*, and *Gambling Activity* were all negative relationships; see Table 1, ows H2a, H2d, H2e, and H2f. Independent variables *Age*, *PG F* + *F*, and *PGSI* all displayed positive relationships to dependent variable *Slots Prop*; see Table 1, rows H2b, H2c, and H2g, respectively.

All hypotheses related to the dependent variable *Slots Prop* were supported with the exception of problem gambling score, measured by the PGSI; contrary to expectations, increased PGSI score was associated with increased support for the proposal to limit access to slot machines, although the overall effect was very small (β_2015_ = 0.05; β_2019_ = 0.055). Overall, however, the patch coefficients for predictors of opinion of proposal to limit access to slot machines were notably higher than for opinion of the Finnish System, with several having β values of 0.3 or higher.

Statistically significant differences between approval of slot machine proposal in 2015 and 2019 datasets were observed for: *Age*, *PG F* + *F*, and *Gender (male)*. In all cases the relationships observed in 2015 were stronger than in 2019, the first two becoming more negative and the latter more positive. See Table 1 for full details.

Finally, approval of the proposal to limit access to slot machines was higher in 2019 (52.3%) than in 2015 (37.2%). However, overall opinion of the Finnish system was lower in 2019 (71.9%) compared to 2015 (77.8%). Using the 2015 and 2019 datasets, the model explained 4.9% and 0.17% of variance (*r*^2^) of opinion of the Finnish system (*Finn Sys*), respectively, with this difference found to be statistically significant (*R*^2^_2015-2019_ = 0.031, *p* < 0.001). The model explained 26.7% and 22.4% of variance in support for the proposition to reduce access to slot machines (*Slots Prop*), respectively, with this difference found to be statistically significant (*R*^2^_2015-2019_ = 0.043, *p* = 0.005).

All hypotheses and results are presented in Table 1, below, for ease of reference.

## Discussion

This work used PLS-SEM multigroup analysis to examine public attitudes toward the Finnish state-monopoly system for regulating gambling, specifically its suitability for achieving the stated purpose of harm reduction and prevention. Results showed that positive attitudes toward the state monopoly decreased between 2015 and 2019, while support for a proposal to reduce the number of slot machines in public spaces increased. A statistically significant change in *R*^2^ of these two items between 2015 and 2019 demonstrates that there was a meaningful shift in public opinion, although the *R*^2^ value for *Finn Sys* itself was very low. In addition, a number of statistically significant predictors of public attitude were identified for each dependent variable.

The results regarding increasingly negative attitudes toward the Finnish system as a whole and increasing support for the proposal to limit access to slot machines may be explained by the fact that there has been heightened awareness of gambling harms in Finnish society and of the ethical issues associated with Veikkaus Oy [6]. As such, the Finnish authorities’ justification for the merger as enhancing capacity to address gambling harm does not appear to be reflected in public opinion.

In addition to increasing the effectiveness of the state monopoly in addressing the harms of gambling, the merger was also presented as enhancing both the services Veikkaus offers and, to a lesser degree, consumer protection practices [8]. Once again, this reasoning does not appear to be reflected in public opinion as several predictors associated with participation in gambling (*ATGS*, *Online Gambling*, *PGSI* score) showed statistically significant negative changes, albeit the overall effect sizes remained small or negligible. Conversely, several predictors’ negative relationship to opinion of the Finnish system was reduced after the 2017 merger, however, the overall effects were small in size and remained negative.

Finally, as part of the restructuring of the Finnish system in 2017 strategies for enhancing harm minimisation, harm prevention, and treatment were developed and implemented [1, 37, 38]. One particularly novel approach has been that the voices of persons with lived experience with problem gambling have been promoted and their stories of recovery have been increasingly shared in public. These previously unheard or marginalised voices may have impacted attitudes between 2015 and 2019, increasing visibility of the harms of gambling and, consequently, reducing positive opinion of the Finnish monopoly system and increasing support for more restrictive measures around gambling.

To date, there is somewhat mixed evidence concerning the effectiveness of public campaigns addressing gambling, specifically, those campaigns funded by industry have not been found to meet the claimed impact [39]. It is likely that public campaigns raising awareness about gambling and it’s potential consequences would benefit from moving away from messages centred on individual responsibility and more focusing on the activities and products associated with gambling. Such campaigns could adopt lessons from other areas of public health [40, 41], or from other areas of political or corporate messaging which have proven to be effective means of influencing public opinion [42–44].

Slot machines (EGMs) have been recognised as one of the most addictive forms of gambling, and historically their prevalence in Finland has been very high [14, 45]. This work hypothesised that positive attitudes to gambling would be associated with reduced support for the proposal to reduced access to slot machines. This hypothesis was supported for both those that endorsed the statement “*gambling is a serious problem in Finland*”, and for positive attitudes toward gambling as measured by the ATGS. However, after the 2017 merger the degree of negativity increased in the former and decreased in the latter; it may be that the increasingly negative tone surrounding public discussions of gambling have changed attitudes towards activities particularly associated with harm in the Finnish context, rather than gambling in general.

Given that overall views of the Finnish system are becoming less positive, the targeted policy action of reducing the number of slot machines available may potentially serve as tangible evidence of attempts to reduce harm, thereby increasing positive attitudes to the system as a whole. However, such a causal relationship cannot be determined from the currently available data and, therefore, would require dedicated research. The complexities of support for gambling may also be a factor; the relationship between positive views of gambling and views on government policy action may not have a black and white relationship. These results, therefore, highlights the importance of including context-specific items in order to assess attitudes to local governance systems, rather than relying on more general measures.

This perspective is supported by the very low R2 value for *Finn Sys*, meaning that the model explained little of the variance in opinions of the Finnish System as a suitable means of reducing harm. This finding is at odds with prior research [22], and suggests that either models should incorporate additional items such as socio-political attitudes to governance, or that attitudes are formed differently according to local context. For example, Finns have traditionally been used to state monopolies, with the gambling market being a particularly special case [8], indeed although opinion of the Finnish system is declining, it is still relatively high at approximately 72%. Further research is needed in this area to build upon existing research and develop appropriate items to predict public attitudes to gambling regulation, for example: cultural acceptance [46, 47], political orientation or attitude [48], risk orientation [49], trust in authority (e.g., [50, 51]), religious affiliation or orientation [52, 53], and so on.

### Limitations

There first limitation of this work is that although it concerns potential changes in attitude between 2015 and 2019, the datasets were not longitudinal. Instead, they were population level surveys with no guarantee that the same individuals completed each of the two surveys. However, response rates were above the international average and the sample size is large enough to reliably identify changes in public attitudes [54]. Similarly, while the merger in 2017 occurred directly at the mid-point between the two surveys, it is unlikely to be the only influence on attitudes and opinions.

Second, attitudes to the Finnish system and the proposal to limit access to slot machines were measured with single items, not via composite measures or any established instruments. In addition, this analysis was conducted using both ATGS and PGSI as continuous scales, rather than applying categories based on respective scores, while this approach is not unusual it is not how these instruments were designed.

Finally, several meta-analyses and reviews [55, 56] have highlighted the fact that psychiatric and behavioural disorders are linked with gambling severity. However, due to the fact that relevant instruments were not included in the original surveys, these factors were not included in our analyses. The need to include more items in research models examining public attitudes is demonstrated by the fact that despite a large number of predictors, our model explained only around 5% variance in attitudes to the Finnish monopoly system.

## Conclusion

This research found that despite a restructuring of the Finnish monopoly system in 2017, public opinion of its suitability for addressing gambling harms declined between 2015 and 2019. Conversely, support for a proposal to limit access to slot machines in public spaces grew during the same timeframe. Additionally, this research identified several statistically significant predictors of public opinion of the Finnish system and for the specific policy action described. While these predictors were based on prior research from the field, the majority were of marginal to small effect sizes. As such, it is likely that context-specific predictors should be included in models, in order to reflect the socio-cultural history of the population being investigated. Such predictors should be determined in respect to the population of interest but, for example, could include items measuring trust in authority, political orientation, cultural acceptance of gambling, or religious affiliation.

Further research is also needed in order to assess the specific relationship between attitudes to gambling in general, regulatory systems, and specific policy actions. It may be that public opinion is more effectively influenced by specific, targeted policies than by institutional or organisational changes. Alternatively, the somewhat mixed messaging behind the justification of the merger may have increased critical attitudes toward the monopoly provider.

Finally, public opinion may have been influenced by increasingly critical discussion of the state monopoly provider in national media, rather than the merger itself. It is possible that campaigns to raise awareness of potential gambling harms may have also served to cast doubt on the ability of the current system to effectively manage those harms. Accordingly, such campaigns would benefit from a more holistic approach to design and dissemination of information.

## Data Availability

The Finnish Gambling data sets (2015 & 2019) are openly available for research purposes from the Finnish Social Science Data Archive (https://www.fsd.uta.fi/en/).
